# AMPK-dependent and independent actions of P2X7 in regulation of mitochondrial and lysosomal functions in microglia

**DOI:** 10.1186/s12964-018-0293-3

**Published:** 2018-11-20

**Authors:** Ponarulselvam Sekar, Duen-Yi Huang, Shie-Liang Hsieh, Shwu-Fen Chang, Wan-Wan Lin

**Affiliations:** 1 Graduate Institute of Medical Sciences, Taipei Medical University, Taipei, Taiwan; 2 Department of Pharmacology, College of Medicine, National Taiwan University, Taipei, Taiwan; 3 Genomics Research Center, Academia Sinica, Taipei, Taiwan

**Keywords:** P2X7, AMPK, Mitophagy, Lysosomal biogenesis

## Abstract

**Background:**

P2X7 is ubiquitously expressed in myeloid cells and regulates the pathophysiology of inflammatory diseases. Since mitochondrial function in microglia is highly associated with microglial functions in controlling neuronal plasticity and brain homeostasis, we interested to explore the roles of P2X7 in mitochondrial and lysosomal functions as well as mitophagy in microglia.

**Methods:**

P2X7^−/−^ bone marrow-derived macrophages (BMDM), primary microglia and BV-2 immortalized microglial cells were used to detect the particular protein expression by immunoblotting. Mitochondrial reactive oxygen species (mitoROS), intracellular calcium, mitochondrial mass and lysosomal integrity were examined by flow cytometry. Mitochondrial oxygen consumption rate (OCR) was recorded using Seahorse XF flux analyzer. Confocal microscopic images were performed to indicate the mitochondrial dynamics and mitophagy after P2X7 activation.

**Results:**

In primary microglia, BV-2 microglial cells and BMDM, P2X7 agonist BzATP triggered AMPK activation and LC3II accumulation through reactive oxygen species (ROS) and CaMKKII pathways, and these effects were abolished by P2X7 antagonist A438079 and P2X7 deficiency. Moreover, we detected the dramatic decreases of mitochondrial OCR and mass following P2X7 activation. AMPK inhibition by compound C or AMPK silencing reversed the P2X7 actions in reduction of mitochondrial mass, induction of mitochondrial fission and mitophagy, but not in uncoupling of mitochondrial respiration. Interestingly, we found that P2X7 activation induced nuclear translocation of TFEB via an AMPK-dependent pathway and led to lysosomal biogenesis. Mimicking the actions of BzATP, nigericin also induced ROS-dependent AMPK activation, mitophagy, mitochondrial fission and respiratory inhibition. Longer exposure of BzATP induced cell death, and this effect was accompanied by the lysosomal instability and was inhibited by autophagy and cathepsin B inhibitors.

**Conclusion:**

Altogether ROS- and CaMKK-dependent AMPK activation is involved in P2X7-mediated mitophagy, mitochondrial dynamics and lysosomal biogenesis in microglial cells, which is followed by cytotoxicity partially resulting from mitophagy and cathepsin B activation.

**Electronic supplementary material:**

The online version of this article (10.1186/s12964-018-0293-3) contains supplementary material, which is available to authorized users.

## Background

Neuroinflammation plays a determinant role in the progress of neurodegeneration [1]. Accumulated findings reveal that in response to brain injury or immunological stimuli, microglial cells become activated and migrate to the site of injury and secrete numerous chemokines, reactive oxygen species (ROS), and pro-inflammatory cytokines. Thus, microglial activation-associated inflammatory response is believed to be the major cause to induce progressive neuronal death [2, 3]. Recently, impaired mitochondrial function resulting from inflammatory and metabolic stresses has been implicated in numerous neurodegenerative diseases [4]. Damage to mitochondria not only causes defects in energy generation, but also triggers ROS production and organelle fragmentation, leading to neuronal cell death [5]. Thus control of mitochondrial quality mostly based on a balance between biogenic renewal and mitophagic culling is crucial. Mitochondrial dynamics including mitochondrial fission and fusion regulates mitochondrial homeostasis. Mitophagy, a selective autophagy that specifically eliminates damaged mitochondria, is a key mechanism to maintain the overall quality of mitochondria [6]. Aberrant mitophagy has been linked to cell death and various neurodegenerative diseases [7].

AMPK is a heterotrimeric conventional serine/threonine kinase that acts to maintain metabolic pathways and cellular energy homeostasis by switching off energy-consuming pathways but stimulating energy production [8]. Increased AMPK activity generally is associated with the phosphorylation at its Thr172 residue in the activation loop by upstream kinases. Studies reveal that AMPK activity is linked to mitochondrial integrity and involves in mitochondrial fission [9] and mitophagy [10]. In addition, AMPK signaling is identified to mediate lysosomal biogenesis and remodeling, which is responsible for digesting cargo acquired during autophagy [11]. Moreover, mTOR acts as an antagonistic and balanced regulator of AMPK-dependent mitophagy and lysosomal biogenesis [11–13]. The latter action is through modulating the major transcription factor TFEB that governs lysosome gene expression [13, 14].

P2X7 is a ligand-gated ion channel receptor, which is ubiquitously expressed and in particular abundantly in myeloid cells. Higher concentrations of extracellular ATP accumulated at sites of tissue injury and inflammation can activate P2X7, leading to numerous cellular events [15]. These include potassium efflux, calcium influx, ROS production, NLRP3 inflammasome activation, decrease of mitochondrial membrane potential, and eventually cellular death [16–19]. Increasing evidence indicates that pro-inflammatory and pro-apoptotic actions elicited by P2X7 contribute extensively to chronic inflammation and pathogenesis of various diseases including the CNS and cardiovascular diseases [20, 21]. Indeed, over the past years emerging data have generated scientific interest focusing on the potential role of P2X7 in microglial activity. P2X7 in microglia has been considered as a drug target for CNS disorders [22] and the brain-penetrant P2X7 antagonists have been in vivo validated on neuropsychiatric disorders [23], drug-resistant epilepsy [24], neuropathic pain [25] and brain tumors [26]. More recently we and other groups observed the actions of P2X7 to induce autophagy [27, 28] and impair lysosomal function in microglia [29]. Despite P2X7 has been studied extensively as an appealing target for neuronal disorders, the detailed signaling and molecular events underlying P2X7-mediated autophagy, regulation of mitochondrial biogenesis and lysosomal remodeling remain unclear. We therefore, in this study investigated the roles of P2X7 in mitochondrial and lysosomal biogenesis for microglial activity, and explored the potential signaling pathways involved.

## Methods

### Animals

WT (C57BL/6) mice were purchased from Laboratory Animal Center, National Taiwan University. The P2X7^−/−^ mice on the C57BL/6 background were obtained from Jackson Laboratory (Maine, US) [30]. All animals were bred under specific pathogen-free conditions in the Laboratory Animal Center, National Taiwan University College of Medicine (Taipei, Taiwan).

### Cell culture

BV-2 cells, a murine immortal microglial cell line generated by infecting primary microglial cells with a v-raf/v-myc oncogene carrying retrovirus (J2) [31], were cultured in complete high glucose DMEM (containing 4 mM L-glutamine and 25 mM glucose) supplemented with 10% fetal bovine serum (FBS), 2 mM L-glutamine, 3.7 g/l NaHCO_3_, 100 U/ml penicillin and 100 μg/ml streptomycin [27]. Primary microglial cells were isolated from cortex and hypothalamus of 1~ 3-day old neonatal WT or P2X7^−/−^ mice as described previously [32]. Briefly mixed type cells were maintained at 37 °C in a humidified incubator containing 5% CO_2_. After 14–16 days, confluent cultures of mixed glia were shaken for 2 h at 180 rpm in a rotary shaker. The detached microglia cells were seeded in DMEM/F12 medium supplemented with 10% FBS, 100 U/ml penicillin, and 100 μg/ml streptomycin at a density of 2 × 10^5^ cells per well in 12-well plates. The purity of microglia cultures was assessed via flow cytometry using CD11b antibody, and more than 95% of sorted cells were CD11b positive. Bone marrow-derived macrophages (BMDM) were cultured as we previously described [33].

### Reagents and antibodies

ATP disodium salt hydrate, BzATP [2′(3′)-*O*-(4-benzoylbenzoyl) adenosine-5′-triphosphate tri(triethylammonium) salt], A438079 hydrochloride hydrate, mitoTEMPO, Compound C, STO-609, oligomycin, carbonyl cyanide-p-trifluoromethoxyphenylhydrazone (FCCP), rotenone, 3-(4,5-dimethyl-2-thiazolyl)-2,5-diphenyl-2H-tetrazolium bromide (MTT), protease inhibitor cocktails and CaMKKβ antibody were from Sigma-Aldrich (St. Louis, MO, USA). Nigericin (Cat. tlrl-nig) was from Invivogen (San Diego, CA, USA). CA-074Me and antimycin A were purchased from Merck Millipore (Massachusetts, USA). MitoSOX Red, Fluo 3-AM, MitoTracker green and LysoTracker Red were from Molecular Probes (Eugene, OR, USA)**.** A769662 was from Calbiochem (San Diego, CA, USA). LC3 antibody was from MBL International (Woburn, MA, USA). Specific antibodies against AMPKα, Drp-1, PINK1, Parkin, phosphorylated forms of AMPKα (T172), mTOR (S2448), CaMKK2 (Ser511), Drp-1 (S637), and anti-Tom 20 were from Cell Signaling Technology (Danvers, MA, USA). Antibody against β-actin (MAB1501) was from Upstate Biotechnology (Charlottesville, VA, USA). Antibody against TFEB was from Bethyl Laboratories (Montgomery, TX, USA). DMEM, trypsin-EDTA, penicillin, ampicillin and streptomycin were from Invitrogen (Rockville, MD, USA). The ECL reagent (Western blotting lightening chemiluminescence reagent plus) was purchased from PerkinElmer (Wellesley, MA, USA).

### siRNA transfection

Mouse siP2X7 (Cat no. SC-42576), siAMPK (Cat. no. SC45313) and scramble nonspecific siRNA were purchased from Santa Cruz Biotechnology (Santa Cruz, CA, USA). BV-2 microglial cells at 50% confluence were transfected with 100 nM siRNA by DharmaFECT Transfection Reagents (Dharmacon Research) following the manufacturer’s instruction. After 48 h transfection, cells were treated with indicated drugs and then harvested for analysis.

### Flow cytometric measurement of mitochondrial ROS (mitoROS), intracellular calcium, mitochondrial and lysosomal mass

After treatment with indicated agents, the mitochondrion-specific superoxide (O_2_^−^) and cytosolic calcium concentration were measured by MitoSOX Red (5 μM) and Fluo 3-AM (l μM) respectively as we previously described [34]. Mitochondrial mass was measured by Mitotracker green. Considering the signals of Mitotracker Green might be affected by the metabolic rate of mitochondria, we also measured the response under FCCP treatment. Lysosomal mass was measured by LysoTracker Red DND-99. Fluorescence signals were detected using flow cytometry (FACS Calibur system Franklin Lakes, NJ, USA) and represented as percentages of control group.

### Measurement of mitochondrial oxygen consumption rate

The oxygen consumption rate (OCR) was measured by the extracellular flux analyzer XF24 (Seahorse Bioscience, Houston, TX, USA). BV-2 cells were plated at 4 × 10^5^ cells/well in a Seahorse 24-well V7 microplate (Seahorse Bioscience) and cultured in complete DMEM growth medium for 24 h in a 5% CO_2_ incubator at 37 °C. Then, the medium was removed and cells were incubated in XF assay medium in the absence of NaHCO_3_ and FBS for 1 h at 37 °C in measuring chamber without CO_2_ input. BzATP, nigericin and the mitochondrial complex inhibitors (oligomycin, FCCP, rotenone and antimycin A) were freshly prepared in XF assay media. In some experiments, A438079 (10 μM) was added into wells prior to inserting the plate into the Seahorse XF24 extracellular flux analyzer. After 32 min of measuring the basal respiration, BzATP (200 μM) or nigericin (10 μM) was injected; oligomycin (2.5 μM) was injected into each well at 50 min, followed by FCCP (1 μM) at 74 min, rotenone (2.5 μM) and antimycin A (2.5 μM) at 98 min. OCR was recorded as pMoles per minute, and calculated as percentage of the OCR value before the treatment of tested agents. ATP turnover and respiratory capacity were measured and calculated after the sequential treatments with oligomycin and FCCP as previously described [27]. Averages of three wells were taken per data point. Antimycin A is an inhibitor of ATP synthase, so OCR reduction after antimycin A treatment represents ATP turnover under specific condition. FCCP is an uncoupling agent of electron transport, and can generate a proton efflux to induce the maximum respiration termed as respiratory capacity or uncoupled respiration.

### Mitochondrial imaging

BV-2 cells were initially fixed with 4% paraformaldehyde at 37 °C followed by permeabilization with 0.2% Triton X-100 for 15 min, and blocking by BSA (5%) and normal IgG (1:300) for 1 h. For mitophagy measurement, immunostaining was then performed using primary antibody against Tom-20 or LC3 (Abcam, Cambridge, UK) in 1% BSA overnight at 4 °C. After washing with PBS, cells were incubated with secondary antibody in 1% BSA in PBS for 1 h at room temperature and then mounted with DAPI Fluoromount-G (SouthernBiotech, Birmingham, AL, USA). Images were acquired using a 100 X Plan-Neofluar oil objective of LSM 880 with Airyscan SR microscopy (Carl Zeiss Micro Imaging GmbH, Jena, Germany). The colocalization of TOM20 (marker of mitochondria) and LC3 (marker of autophagosome) was determined by Zen colocalization software and on a pixel by pixel basis. Every pixel in the image was plotted in the scatter diagram based on its intensity level from each channel. The colocalization coefficients were measured for each channel.

### Subcellular fractionation and immunoblotting analysis

After indicated treatment, the medium was aspirated. The nuclear, cytoplasmic and mitochondrial extracts were prepared by hypotonic and Nonidet P40 detergent lysis buffer as previously described [33]. Briefly, cells were washed with ice-cold PBS, and then lysed in hypotonic buffer (10 mM HEPES pH 7.9, 10 mM KCl, 1.5 mM MgCl_2,_ 0.34 M sucrose, 10% glycerol, 0.1% Triton X-100, 1 mM DTT, 0.1 mM PMSF) supplemented with proteinase inhibitors for 5 min on ice. The lysates were spun down at 160 g, 4 °C for 15 min. The pellet contains nuclei, and the supernatant contains cytoplasm and mitochondria. The supernatant was further cleared by centrifugation at 16,200 g at 4 °C for 15 min. The supernatant is the cytosolic extract and the pellet is the mitochondrial extract respectively. The pelleted nuclei were washed with hypotonic buffer, and then resuspended in nuclear lysis buffer (20 mM HEPES pH 7.9, 300 mM KCl, 0.1% Triton X-100, 0.5 mM EDTA, 0.1 mM PMSF, 1 mM DTT) containing proteinase inhibitors. Nuclei were then homogenized on ice for 30 min using a dounce homogenizer for 20 strokes. The supernatant (nuclear extract) was collected by centrifugation at 24,500 g for 15 min. Both cytoplasmic and nuclear extracts were dialyzed against D100 buffer (20 mM HEPES pH 7.9, 100 mM KCl, 0.2 mM PMSF). Protein levels were determined by standard immunoblotting as previously described [33].

### Intracellular cathepsin B activity assay

Intracellular active cathepsin B released from destabilized lysosomes was determined by the MR cathepsin detection kit (Part #937; ImmunoChemistry Technologies, Bloomington, MN, USA) as previously described [35]. The cresyl violet fluorogenic cathepsin B substrate MR-RR_2_ is cell membrane permeable and can detect active cathepsin B enzyme in intact cells. After hydrolyzing by cathepsin B, the substrate is converted to the red fluorescent form. After drug treatment, BV-2 cells were centrifuged at 100 g for 5 min, re-suspended in 1 ml fresh medium, and treated with cathepsin B substrate, which was reconstituted with ddH_2_O to make a reagent solution in the ratio of 1:10. After incubating at 37 °C for 2 h, cells were washed twice with PBS and then immediately submitted to fluorescence spectrophotometer (Thermo Scientific Varioskan® Flash).

### Cell viability assay

Cells after drug treatment were incubated with MTT (5 mg/ml) for 1 h at 37 °C. The supernatants were aspirated, and the formazan granules generated by the live cells were dissolved in DMSO. The OD values at 550 and 630 nm were measured by use of a microplate reader. The net absorbance (OD550–OD630) indicating the enzymatic activity of the mitochondria and implying cell viability was represented as 100% of the individual control.

### Statistical analysis

Data were expressed as mean ± S.E.M. Multiple groups were compared by one-way analysis of variance and Bonferroni post-test, making use of Graph pad software (Graph Pad Software, San Diego, CA, USA). Two groups were compared with an unpaired Student’s *t* test and two-tail *p* value. Results were considered statistically significant when *p* < 0.05.

## Results

### P2X7 induces Ca/CaMKK- and ROS-dependent AMPK activation in microglia and macrophages

**Fig. 1:**
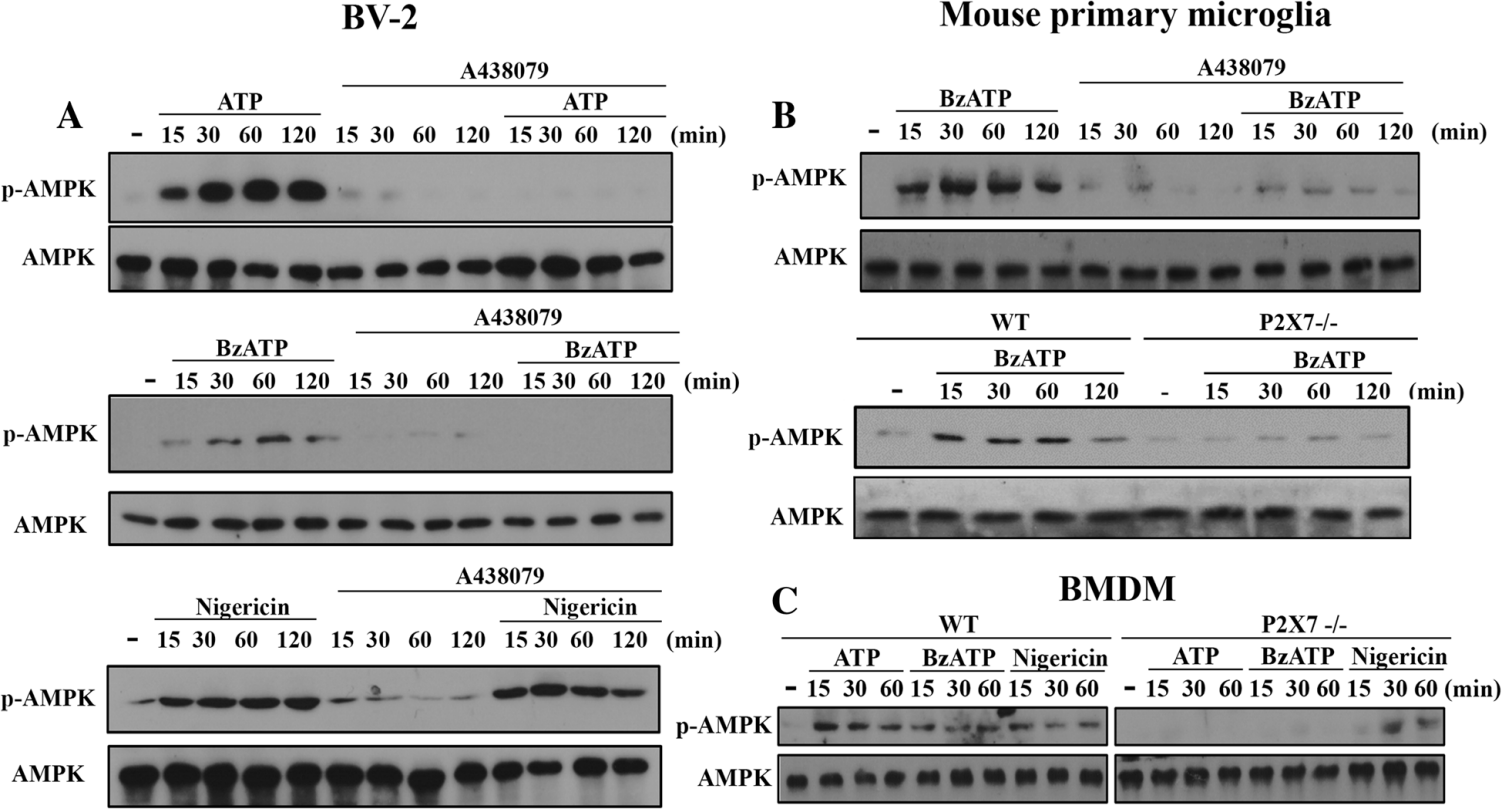
P2X7 induces AMPK activation in BV-2, primary microglia and BMDM. BV-2 cell line (**a**), mouse primary microglia (**b**) and BMDM (**c**) isolated from WT or P2X7^−/−^ mice were treated with ATP (1 mM), BzATP (200 μM), or nigericin (10 μM), either with or without the pre-treatment of A438079 (10 μM) for 30 min. After treatment for indicated time intervals, total cell lysates were collected for immunoblotting. Data were representative of 3 independent experiments. Data quantification was shown in Additional file 1: Figure S1

In order to link the energy-sensing kinase AMPK and P2X7 signaling, we first determined the effects of two P2X7 agonists, ATP and BzATP, on AMPK activation. In BV-2 microglia, we found a time-dependent and rapid AMPK phosphorylation upon agonist treatment. A438079, the P2X7 competitive antagonist, can abolish ATP- and BzATP-induced AMPK phosphorylation, but not that induced by nigericin, which is a potassium ionophore and exerts some actions mimicking P2X7 such as NLRP3 inflammasome activation via K^+^ efflux (Fig. 1a). Similar to BV-2 cells, BzATP also activated AMPK in mouse primary microglial cells (Fig. 1b) and BMDM (Fig. 1c), and this effect of BzATP was also abolished by P2X7 knockout (P2X7-/-) (Fig. 1b and c) and/or A438079 (Fig. 1b). In contrast, nigericin-induced AMPK activation in BMDM was unaltered by P2X7 knockout (Fig. 1c). All blotting data were quantified and shown in the Additional file 1: Figure S1.

**Fig. 2:**
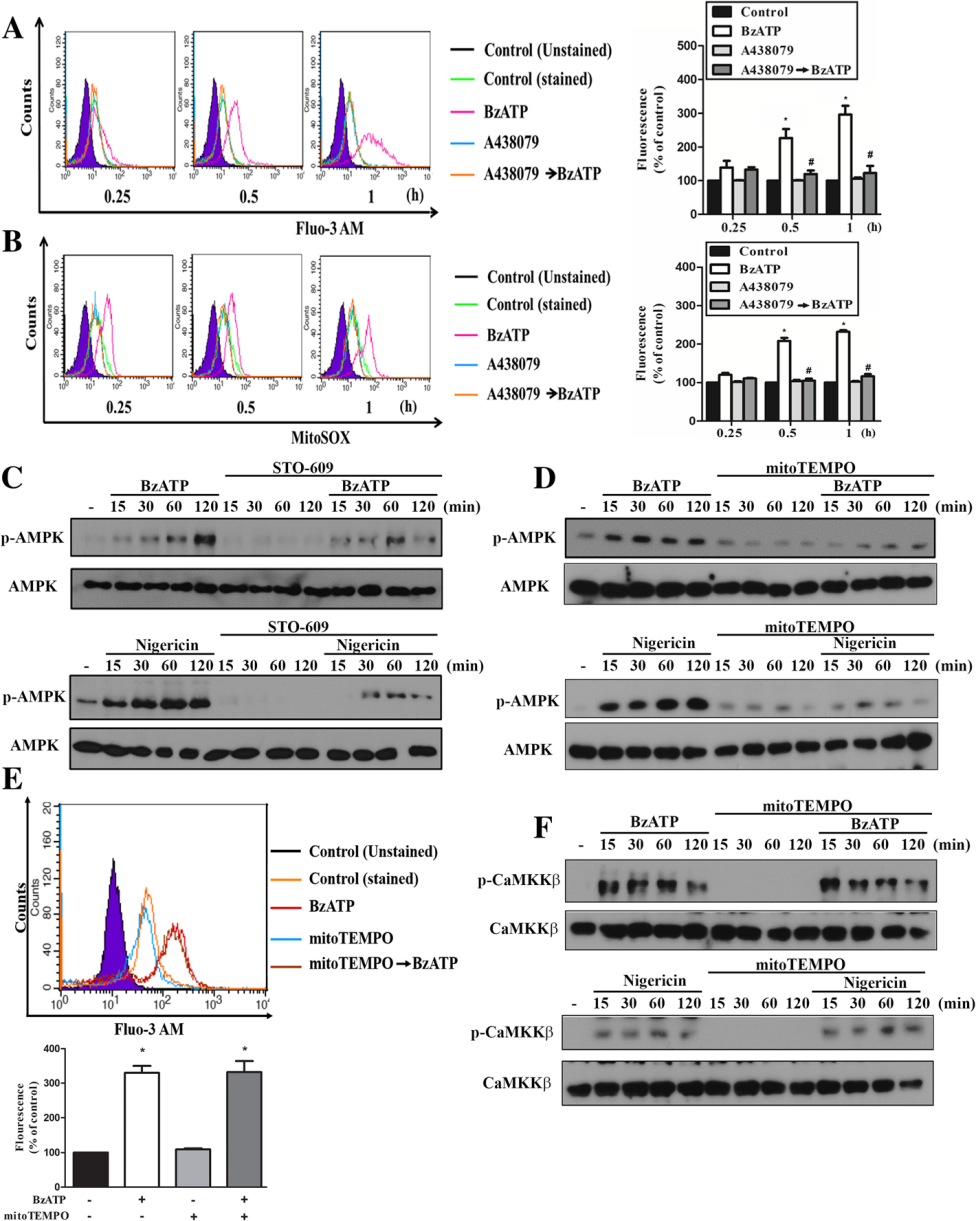
P2X7-mediated AMPK activation depends on mitoROS and Ca/CaMKK in BV-2 cells. BV-2 cells were pretreated with A438079 (10 μM) for 30 min, followed by the stimulation with BzATP (200 μM) for 15, 30 or 60 min. Then Fluo-3 AM (1 μM) (**a**) or MitoSOX (5 μM) (**b**) were used to determine cytosolic calcium and mitochondrial ROS, respectively. The fluorescence intensity was presented as percentages of the control group without any treatments. Data were the mean ± S.E.M. of at least 3 independent experiments. *p < 0.05, indicating the significant stimulating effect of BzATP; #*p* < 0.05, indicating the blockade effect of A438079. In **c**-**f** cells were pretreated with STO-609 (10 μM) (**c**) or mitoTEMPO (250 μM) (**d**-**f**) for 30 min, followed by the treatment with either BzATP (200 μM) or nigericin (10 μM) at the indicated time points. Cell lysates were used for immunoblotting (**c**, **d** and **f**) and in some experiments cytosolic calcium level was determined (**e**). Data were representative of 3 independent experiments. *p < 0.05, indicating the significant effects of BzATP; #*p* < 0.05, indicating the antagonist effect of A438079 and mitoTEMPO on the effects of BzATP. Data of **a**, **b** and **e** were the mean ± S.E.M. from 3 independent experiments. Data quantifications of **c**, **d** and **f** were shown in Additional file 2: Figure S2

Next, we assessed the signaling events underlying P2X7-mediated AMPK activation. Since increases of intracellular calcium and ROS production upon P2X7 activation have been demonstrated in neurons [36] and microglial cells [37], we sought to determine whether Ca^2+^/calmodulin-dependent protein kinase kinase (CaMKK) and ROS involve in P2X7-mediated AMPK phosphorylation. Using Fluo-3 AM and mitoSOX, we found that [Ca^2+^]i and mitoROS levels were significantly and rapidly increased by BzATP within 60 min treatment, and both events were abolished by A438079 (10 μM) (Fig. 2a, b). We further used STO-609, a specific inhibitor of CaMKK, to determine the involvement of CaMKK in AMPK signaling. As result shown in Fig. 2c, BzATP- and nigericin-induced AMPK phosphorylation were inhibited in BV-2 cells pretreated with STO-609 (10 μM). Likewise, we found that the presence of mitoTEMPO, an inhibitor of mitoROS, can abrogate AMPK phosphorylation caused by BzATP and nigericin in BV-2 cells (Fig. 2d). To determine whether mitoROS might regulate Ca/CaMKK signaling, we treated cells with mitoTEMPO and found that there were no significant alterations on the increased [Ca^2+^]i (Fig. 2e) and the phosphorylation status of CaMKK (Fig. 2f) induced by BzATP or nigericin. All these results suggest that CaMKK and mitoROS may contribute independently in P2X7-mediated AMPK activation. All blotting data of Fig. 2c, d and f were quantified and shown in the Additional file 2: Figure S2A, B and C, respectively.

### P2X7 induces AMPK-dependent mitochondrial fission and mitophagy

**Fig. 3:**
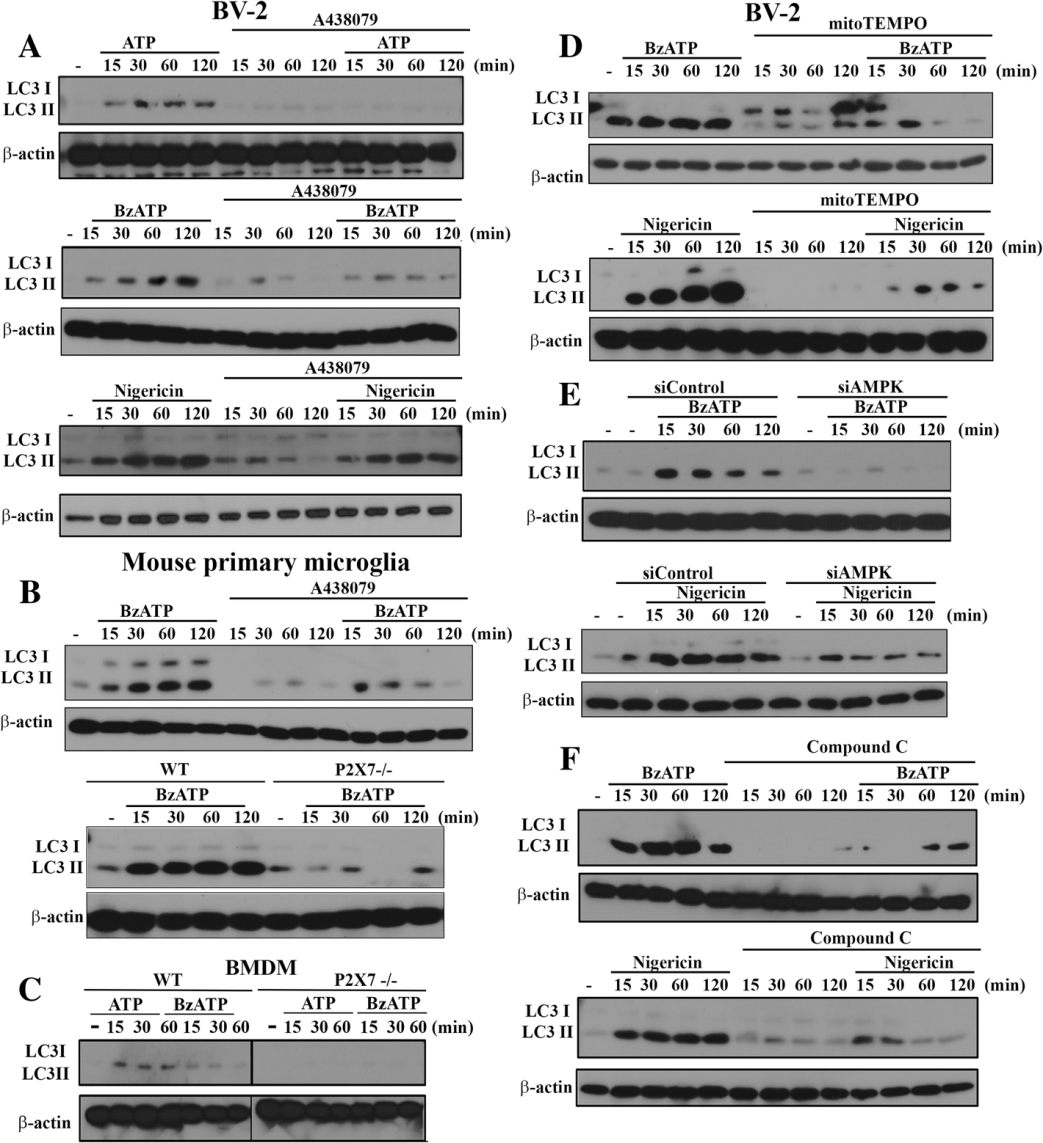
AMPK activation and mitoROS production mediate LC3II accumulation. BV-2 cells were pre-treated with A438079 (10 μM, **a**), mitoTEMPO (250 μM, **d**) or compound C (10 μM, **f**) for 30 min, or siAMPK (100 nM, **e**) for 48 h. Then cells were treated with ATP (1 mM), BzATP (200 μM), or nigericin (10 μM) for the indicated time points. In some experiments, mouse primary microglia (**b**) and BMDM (**c**) isolated from WT and P2X7^−/−^ mice were treated as indicated. Total cell lysates were collected for immunoblotting. Data were representative of 3 independent experiments. Data quantification was shown in Additional file 3: Figure S3

To further reveal the consequence of P2X7-activated AMPK signaling on mitochondria function, we analyzed the mitochondrial dynamism and mitophagy processing. To assess mitophagy, we first determined LC3II, whose accumulation is an index of autophagy [38]. We found that both BzATP and ATP can increase LC3II accumulation in BV-2 (Fig. 3a), primary microglia (Fig. 3b) and BMDM (Fig. 3c), and these effects were greatly attenuated by A438079 and/or P2X7 gene knockout. In contrast, A438079 cannot abolish the LC3II accumulation triggered by nigericin (Fig. 3a). In addition, in the situation with mitoTEMPO treatment (Fig. 3d), AMPK silencing (Fig. 3e) or treatment with AMPK inhibitor compound C (Fig. 3f), BzATP- and nigericin-induced LC3II increases in BV-2 cells were greatly inhibited. These findings further emphasize the AMPK-dependence of autophagy/mitophagy induction. All blotting data of Fig. 3 were quantified and shown in the Additional file 3: Figure S3.

**Fig. 4:**
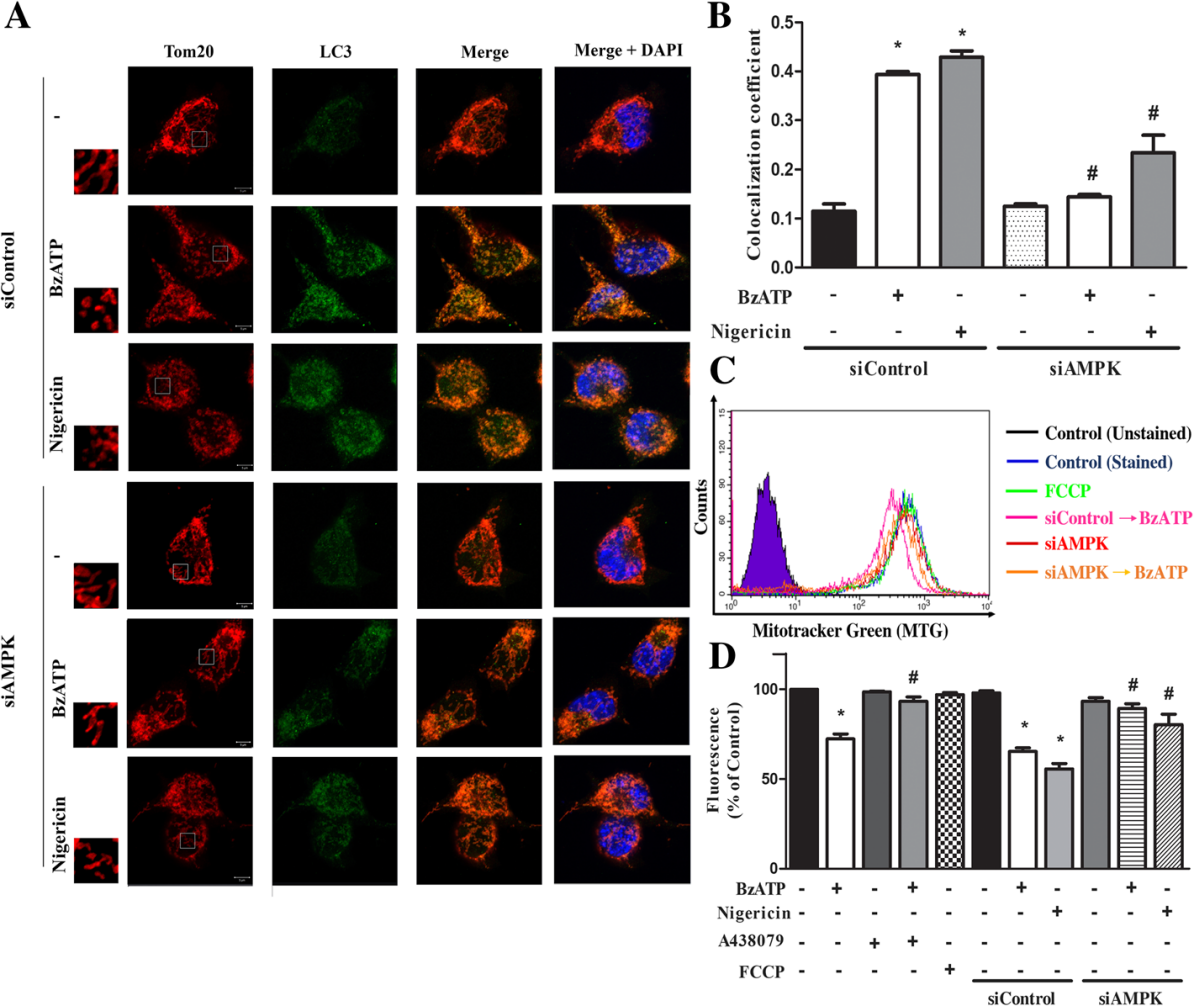
P2X7 induces mitophagy via AMPK activation. **a** BV-2 cells were silenced by siRNA against AMPK, and then stimulated with BzATP (200 μM) or nigericin (10 μM) for 1 h. Afterwards cells were stained with LC3 (indicator of autophagosome) and Tom 20 (indicator of mitochondria). The co-localization of LC3 and Tom 20 was quantified by Zen colocalization module program (**b**). In **c** and **d**, after silencing of AMPK or pretreatment with A438079 (10 μM), BV-2 cells were stimulated with BzATP, nigericin or FCCP (1 μM) for 2 h, and mitochondria mass was determined by mitotracker green. Data were the mean ± S.E.M. from 3 independent experiments. *p < 0.05, indicating the significant effects of BzATP and nigericin; #*p* < 0.05, indicating the blockade effect of A438079 or siAMPK

Mitochondrial dynamics upon P2X7 activation was analyzed by confocal laser scanning microscopy with specific antibodies against Tom 20 and LC3. Tom-20 is a subunit of the translocase of the mitochondrial outer membrane (TOM) complex, and becomes a common marker of mitochondria. Its co-localization with LC3II indicates the formation of mitophagy. Our data revealed a higher mitochondrial fission upon BzATP or nigericin treatment, and the concomitant co-localization of LC3 with Tom 20 (Fig. 4a). These effects of BzATP and nigercin were markedly attenuated by AMPK silencing (Fig. 4b). Along with the changes of mitochondrial dynamics, the fluorescence of Mitotracker green (an index of mitochondrial mass) was reduced upon BzATP or nigericin treatment for 2 h (Fig. 4c, d). Previously some studies concern the action specificity of higher concentrations of Mitotracker green for its action might depend not only on mitochondrial mass but also on metabolic rate [39], thus we determined the response of FCCP, which is a mitochondrial uncoupler. As a result, in our working concentration of Mitotracker green (50 nM), FCCP (1 μM) treatment for 2 h did not affect the fluorescence of mitotracker green (Fig. 4d). Moreover, the action of BzATP was also blocked by A438079 and siAMPK, while that of nigericin was reversed by siAMPK only (Fig. 4d). All these data suggest that P2X7 activated AMPK signaling may mediate mitochondrial fission.

**Fig. 5:**
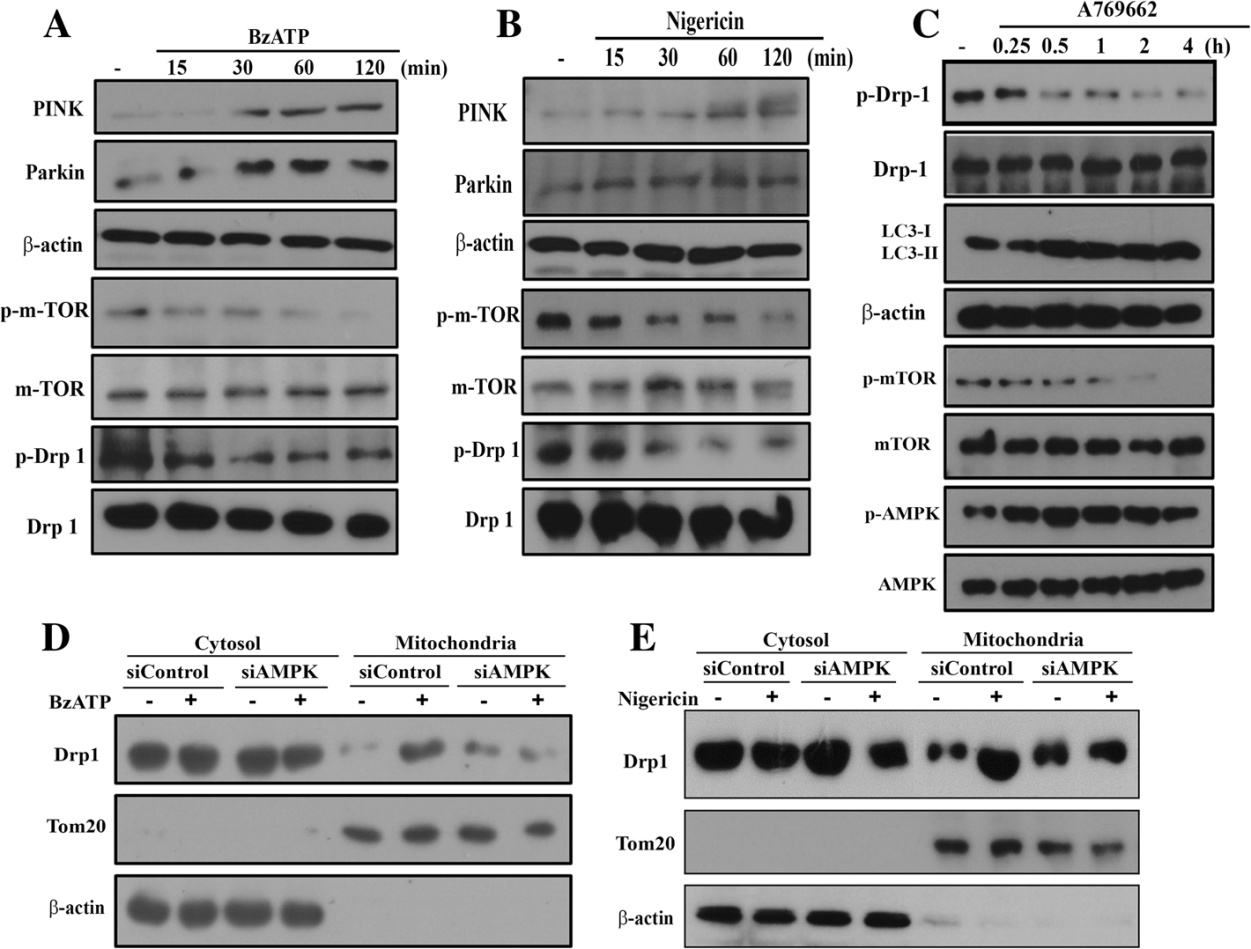
P2X7 upregulates PINK and Parkin, and induces Drp1 translocation to mitochondria. **a-c** BV-2 cells were stimulated with BzATP (200 μM), nigericin (10 μM) or A769662 (20 μM) for indicated time periods. **d** and **e** After AMPK silence, BV-2 cells were stimulated with BzATP (**d**) or nigericin (**e**) for 1 h. Then cellular fractionations of cytosol and mitochondria were prepared. Cell lysates were subjected to SDS-PAGE for immunoblotting. Data were representative of 3 independent experiments. Data quantification was shown in Additional file 4: Figure S4

To further address the underlying basis for P2X7-induced mitophagy, we investigated some essential molecules contributing to mitophagy. As shown in Fig. 5a and b, we found that BzATP and nigericin increased the protein levels of both PINK and Parkin, but inhibited the phosphorylation of mTOR and Drp-1 (Fig. 5a, b). Because Drp-1 phosphorylation at S637 is a negative regulator of mitochondrial fission [40, 41], our findings of reduced Drp-1 phosphorylation support the P2X7-mediated mitochondrial fission. Likewise, A769662 (an AMPK activator) can time-dependently induce AMPK phosphorylation and LC3II accumulation, but decrease mTOR and Drp-1 phosphorylation (Fig. 5c). Moreover, Drp-1 was translocated to mitochondria after BzATP or nigericin treatment for 1 h, and this effect was decreased in cells with AMPK silencing (Fig. 5d, e). All these data together further support the mitophagy induced by BzATP and nigericin is mediated by AMPK activation. All blotting data of Fig. 5 were quantified and shown in the Additional file 4: Figure S4.

### P2X7 inhibits mitochondrial respiration via a pathway independent of AMPK

**Fig. 6:**
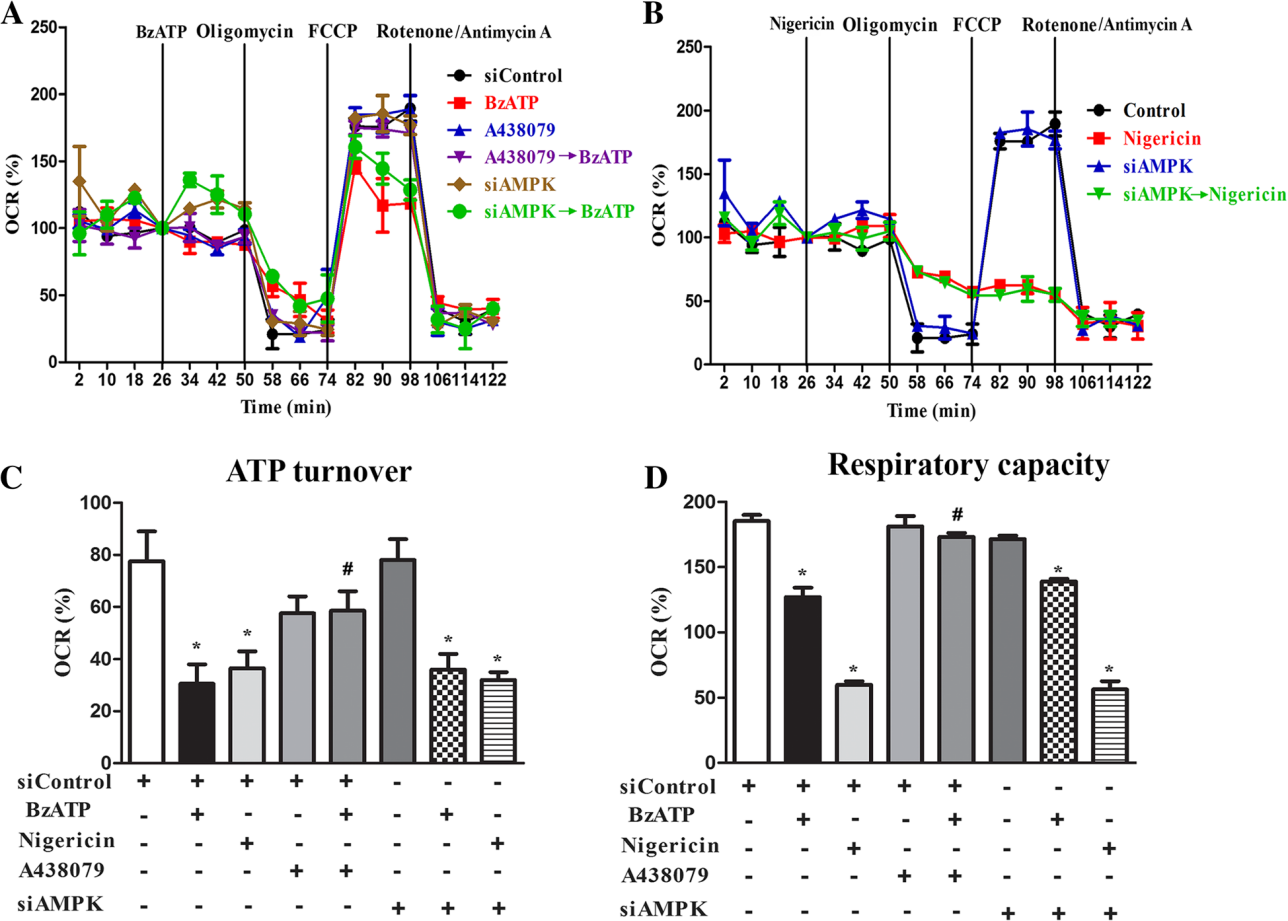
P2X7 activation impairs mitochondrial respiration via an AMPK-independent pathway. **a** and **b** The representative effects of BzATP and nigericin on OCR were shown. BV-2 cells were pretreated with vehicle, A438079 (10 μM) or transfected with siControl or siAMPK before inserting the XF cell culture plate with cells in the Seahorse XF24 analyzer. The cells were treated with BzATP, nigericin from the port A, and subsequently oligomycin (2.5 μM), FCCP (1 μM) and rotenone (2.5 μM) plus antimycin A (2.5 μM) as indicated. OCR was measured as % of the basal respiration value at the time point before giving the major stimulus from port A. **c** and **d** ATP turnover and respiratory capacity were determined from 3 independent experiments in Seahorse XF24 analyzer. *p < 0.05, indicating the significant effects of BzATP and nigericin responses. #*p* < 0.05, indicating the antagonistic effect of A438079 on BzATP response

To further investigate whether mitochondrial respiratory function is affected in parallel with mitochondrial fission and mitophagy triggered by P2X7, we measured the OCR with the Seahorse flux analysis. With sequential addition of several mitochondrial stressors such as oligomycin, FCCP, rotenone and antimycin, we determined ATP turnover and respiratory capacity (or named uncoupled respiration). Our results showed that within the 26 min incubation period, BzATP and nigericin had no significant effects on resting OCR, but both suppressed ATP turnover and respiratory capacity of mitochondria (Fig. 6a, b). Moreover, the effects of BzATP were abolished by the P2X7 antagonist A438079. Notably nigericin treatment led to an enormous decrease of respiratory capacity compared to that stimulated by BzATP (Fig. 6c, d). Next we determined the link between AMPK activation and respiratory dysfunction downstream P2X7 signaling. We found that AMPK silencing did not affect the BzATP- and nigericin-triggered suppression of mitochondrial ATP turnover and respiratory capacity (Fig. 6c, d). These findings indicate an AMPK-independent mechanism in P2X7 signaling to impair mitochondrial respiration.

### P2X7 induces lysosomal biogenesis via AMPK pathway

**Fig. 7:**
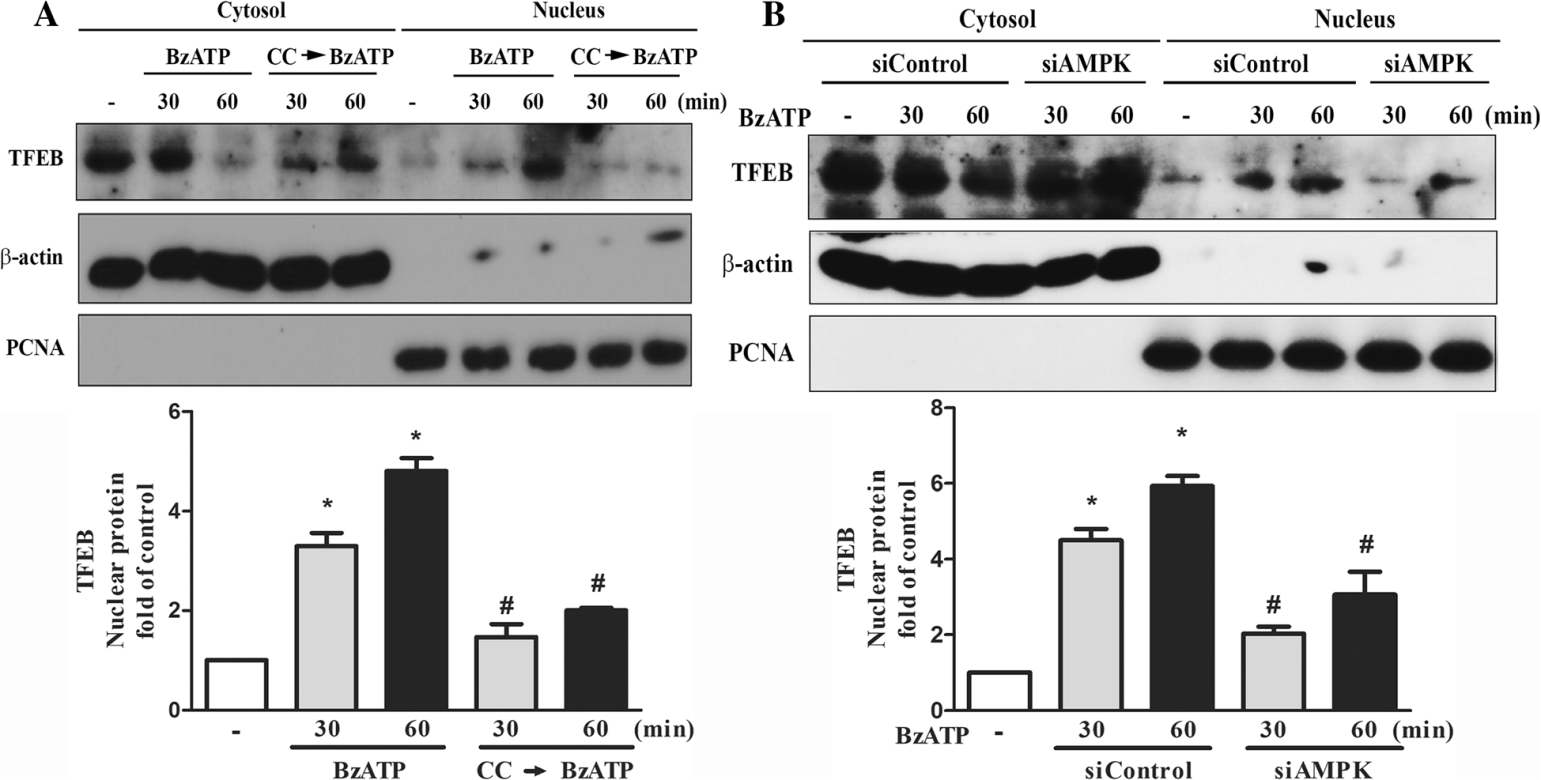
P2X7 induces nuclear translocation of TFEB. After pretreatment with compound C (10 μM) (**a**) or silencing of AMPK (**b**), BV-2 cells were stimulated with BzATP for indicated times. Cell fractionations of cytosol and nuclei were conducted and TFEB protein level was determined by immunoblotting. Data were the mean ± S.E.M. from 3 independent experiments. *p < 0.05, indicating the significant effect of BzATP. #*p* < 0.05, indicating the antagonistic effects of compound C and AMPK silencing on BzATP response

Current understanding in regulation of lysosomal biogenesis and lysosomal function is still limited. To reveal the involvement of P2X7 signaling in the process of lysosomal biogenesis, we measured the TFEB expression and its cellular localization after BzATP treatment. TFEB is the major transcription factor for lysosomal biogenesis and its cellular localization is counter-regulated by AMPK and mTOR [12]. As shown in Fig. 7a, the cytosolic level of TFEB was rapidly decreased along with an increase of TFEB in the nuclei after BzATP treatment. This nuclear translocation of TFEB was reduced by compound C pretreatment (Fig. 7a) or siAMPK (Fig. 7b).

**Fig. 8:**
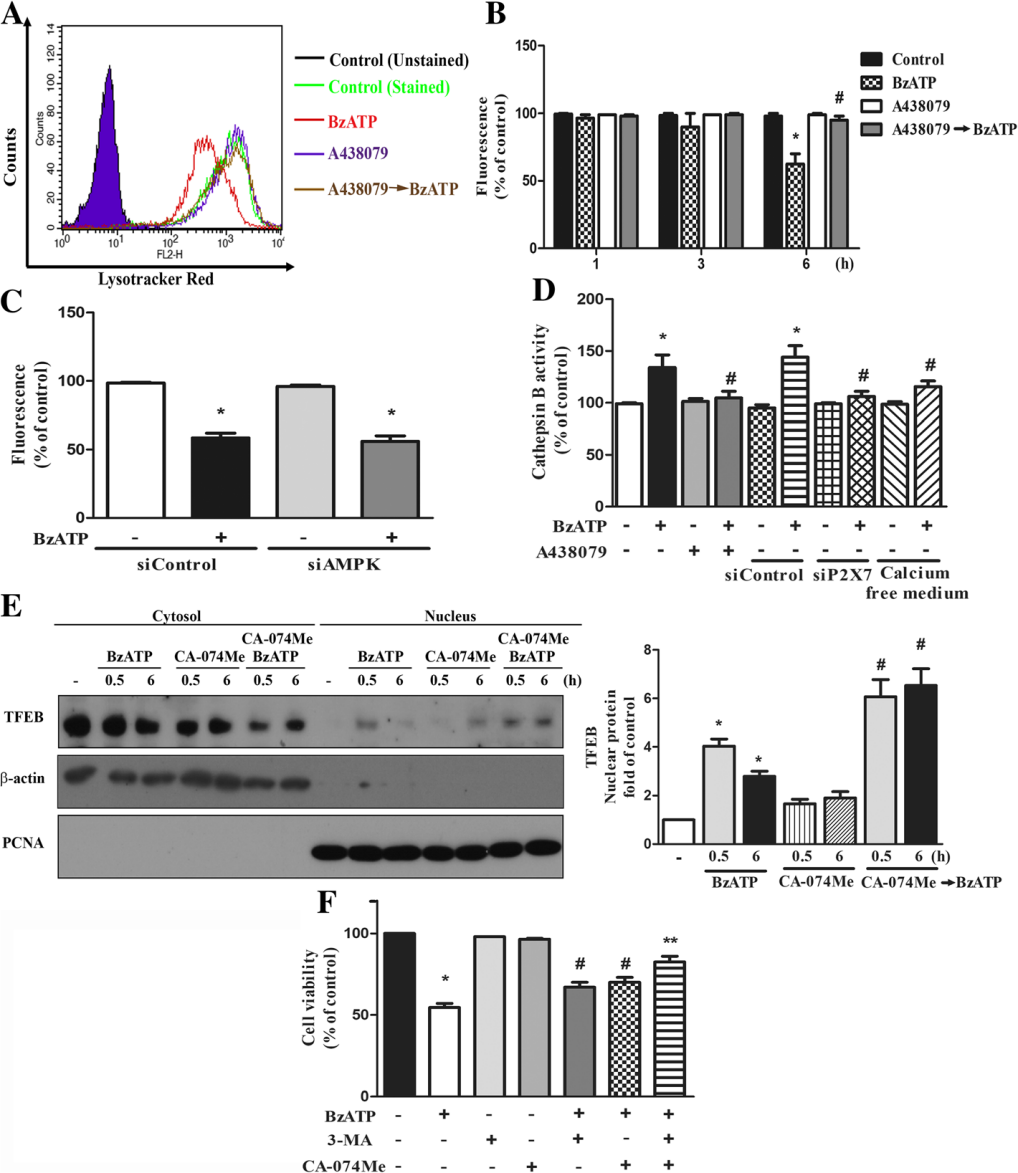
P2X7-induced lysosomal rupture and cathepsin B activation contribute to cell death. **a-c** BV-2 cells were treated with A438079 (**a** and **b**), siAMPK (**c**) and/or BzATP for 6 h. Lysotracker Red staining was determined by flow cytometry. **d** After treatment with A438079, si-RNA against P2X7 or calcium-free medium, BV-2 cells were stimulated with BzATP for 6 h. Intracellular cathepsin B activity was determined using MagicRed cathepsin detection kit and fluorescence spectrophotometer. **e** BV-2 cells were treated with BzATP, either in the absence or presence of CA-074Me (10 μM) for the times indicated. TFEB localization in the cytosol and nuclei was determined. **f** After treatment with BzATP, either in the absence or presence of 3-MA (5 mM) and/or CA-074Me (10 μM) for 6 h, cell viability was determined by MTT assay. Data were the mean ± S.E.M. from 3 independent experiments. *p < 0.05, indicating the significant BzATP responses. #*p* < 0.05, indicating the significant effects of A438079, calcium free medium and CA-074Me  to antagonize or potentiate BzATP response. **p < 0.05, indicating the additive effect of 3-MA and CA-074Me on BzATP-induced cell death

Using Lysotracker Red staining as the index of lysosome stability, we found that BzATP did not change the Lysotracker Red intensity until 6 h, and the inhibitory effect of BzATP at 6 h was prevented by A438079 (Fig. 8a, b) but not by AMPK silencing (Fig. 8c). Accordingly, enzymatic activity of intracellular cathepsin B was increased after BzATP treatment for 6 h and this effect was blocked by A438079, P2X7 silencing or calcium-free media (Fig. 8d). Previously cathepsin B was shown to mediate cell death [42] and negatively regulate TFEB translocation to nuclei [43]. To further understand the consequence of this event under P2X7 activation, we used cathepsin B inhibitor CA-074Me. As shown in Fig. 8e, CA-074Me caused a synergistic effect with BzATP to promote nuclear translocation of TFEB*.* Moreover, our data showed that BzATP-induced decrease of  BV-2 cell viability was reversed by treatment with CA-074Me (Fig. 8f). In addition, because autophagy/mitophagy is a double edged sword on cell fate, we further used 3-MA to determine the role of mitophagy in P2X7-mediated cell death. 3-MA is an inhibitor of class III PI3K and has been used as an inhibitor of autophagy/mitophagy. As a result, our data revealed the ability of 3-MA to reduce BzATP-induced cell death, and combination of CA-074Me and 3-MA had an additive effect (Fig. 8f). These findings suggest the involvements of cathepsin B and mitophagy in P2X7-induced cell death.

## Discussion

Failure of mitophagy is involved in the progression of several neurodegenerative disorders [44, 45]. Therefore, upregulation of mitophagy holds potential for the development of therapeutic interventions towards confronting neurodegenerative diseases in humans. Recently, a few studies demonstrate that P2X7 can modulate autophagic flux in microglia [27, 28], and mitochondrial toxicity is a key event leading to cell death induced by P2X7 activation [27, 46]. Furthermore, P2X7 induces autophagy in myoblasts and myotubes in a mouse model of Duchenne muscular dystrophy, and such autophagic cell death subsequently leads to muscular dystrophy [47]. Induction of autophagy in monocyte/macrophages by P2X7 activation was found to contribute to the bacterial killing [48]. Moreover, P2X7 is suggested to be a key modulator in oxidative stress-mediated autophagy and inflammation in an experimental nonalcoholic steatohepatitis [49] and intestinal inflammation [50]. Of note, P2X7 also induces cellular stress events to impair lysosomal function and cause lysosomal alkalinization, which in turn negatively regulate autophagy [29]. To date the regulatory role of P2X7 activation in lysosome function is much less understood than that in mitochondria. The functional integration between mitochondria and lysosome in mitophagy regulated by P2X7 activation requires further exploration, in particular focusing on the action time course and mediated signaling pathways.

In this study, we found that microglia respond dramatically to P2X7 agonists. After P2X7 activation by either selective agonist BzATP or non-selective agonist ATP, rapid appearances of LC3II accumulation (Fig. 3), mitochondrial fission (Fig. 4a) and decreased mitochondrial mass (Fig. 4c, d) were observed. Meanwhile, several molecular events positively contributing to mitophagy were concomitantly detected, including AMPK phosphorylation (Fig. 1), mitochondrial translocation of Drp-1 (Fig. 5d), increased PINK and Parkin expression (Fig. 5a), and decreased Drp-1 phosphorylation at S637 and mTOR phosphorylation (Fig. 5a). Mechanistically we demonstrate for the first time that AMPK is a key signaling modulator of P2X7 to induce mitophagy, mitochondrial fission and TFEB nuclear translocation in microglia.

AMPK is crucial for mediating mitophagy and modulating mitochondrial dynamics and biogenesis [51]. With regard to mitophagy, AMPK is a key signaling molecule to initiate autophagic flux. In addition, AMPK can regulate PINK (a mitochondrial kinase) and Parkin (an ubiquitin ligase) which are two key factors in mitophagy [52]. Accumulation of PINK on the mitochondrial outer membrane can regulate Parkin for mitophagy. Study further reveals the ability of AMPK to phosphorylate PINK1 at S495, leading to mitophagy [53]. In this study, we demonstrated P2X7 activation can increase PINK and Parkin in microglia cells. In terms of mitochondrial dynamics, AMPK is known to inhibit Drp-1, which is a cytoplasmic guanosine triphosphatase and catalyzes mitochondrial fission by changing its cellular localization, phosphorylation ratio at S616 and S637 [54], or downregulation [55]. Nevertheless, an opposite finding of AMPK to induce mitochondrial fragmentation via phosphorylation of MFF, a mitochondrial outer-membrane receptor for Drp1, is also demonstrated [9]. Our current study apparently supports the roles of AMPK in mediating mitophagy and mitochondrial fission upon P2X7 stimulation of microglial cells. This is because AMPK silencing can reverse the effects of BzATP on LC3II accumulation and translocation to mitochondria, Drp-1 phosphorylation and mitochondrial translocation***,*** and mitochondrial fission.

In addition to involvement in the degradation by forming autophagosome, lysosome is recently recognized as a signaling hub permitting the coordination of several homeostatic signaling pathways [11]. TFEB was recently reported as the major transcription factor to activate lysosome- and autophagy-related genes, thereby increasing the number of lysosomes and promoting autophagy-related cargo degradation [56]. TFEB activity is timely controlled antagonistically by mTOR and AMPK signaling pathways [11–13]. Accordingly, in our study AMPK silencing and compound C treatment can inhibit BzATP-induced TFEB translocation to nuclei (Fig. 7a, b). Despite the enhanced TFEB activation by P2X7 signaling at early stage, longer incubation of BzATP led to an AMPK-independent lysosomal destabilization (Fig. 8c) and cathepsin B release (Fig. 8d)*.* Our data further suggest that cathepsin B release from lysosomes may contribute to cell death caused by P2X7 activated (Fig. 8f). Currently it remains unclear how the integrity of lysosomal membrane is altered, leading to the leakage of lysosomal enzymes to the cytoplasm and triggering cell death.

Although extracellular ATP and BzATP were shown to induce AMPK phosphorylation in MCA38 colon cancer cells [57] and HUVEC cells [58], the upstream signaling pathway remains to be elucidated. Our data revealed that P2X7-induced mitoROS (Fig. 2d) and CaMKK activation (Fig. 2c) are involved in the rapid activation of AMPK. Here we propose that both ROS and Ca^2+^ signals are functionally independent, because mitoTEMPO which is a selective scavenger of mitoROS [59, 60] cannot alter the increased intracellular calcium and CaMKK activation (Fig. 2e, f). Moreover, P2X7-mediated AMPK activation is accompanied by the inhibition of mTOR that is involved in protein translation. All these findings support the notion that ROS and CaMKK function as two major upstream mediators of AMPK activation [61, 62].

Besides timely regulating mitochondrial dynamics and mitophagy, AMPK activity also affects mitochondrial respiration [63]. In our study, we found that P2X7-mediated inhibitions of ATP production and respiratory capacity are AMPK independent. In contrast to the reversal effects as we discussed above, AMPK silencing did not alter BzATP-induced inhibition of mitochondrial respiration. We speculate that this effect might be due to the changes of calcium and potassium homeostasis. Intracellular calcium overload and dramatic potassium efflux upon P2X7 activation might interrupt the mitochondrial membrane potential and cytosolic pH level that may play important roles in maintaining the electron transport efficiency and mitochondrial complex activities [64]. Supporting this notion is our finding that nigericin can induce a higher response than BzATP to decrease uncoupled respiration (Fig. 6d). Thus we suggest that K^+^ efflux induced by P2X7 and nigericin is the potential mediator to impair mitochondrial respiration. In addition, based on the less dependence of nigericin responses on AMPK as compared to BzATP, e.g. loss of mitochondrial mass (Fig. 4d) and induction of Tom-20 and LC3 colocalization (Fig. 4b), K^+^ efflux might exert other direct actions independent of Ca/CaMKK/AMPK to regulate mitochondrial dynamics and mitophagy. Moreover, we also observed the effects of nigericin to increase mitoROS and [Ca^2+^]i in BV-2 cells (data not shown). Therefore, it is suggested that K^+^ efflux itself can indirectly change the intracellular calcium level and mitochondrial function.

In this study we used pharmacologically selective AMPK activator A769662 and genetic silencing approach to demonstrate the role of AMPK in the P2X7 actions. A769662 is the relatively most selective AMPK agonist compared to others like metformin and AICAR. Nevertheless, some recent studies still question the selectivity of A769662 at concentrations above 50–100 μM [63, 65, 66]. In our study, A769662 alone at our working concentration (i.e. 20 μM) did not alter intracellular calcium level in BV-2 cells (data not shown). Moreover, silencing AMPK exerted opposite actions of A769662, in terms of LC3II protein level and phosphorylation of mTOR and Drp-1. Therefore, the involvement of AMPK in P2X7-mediated actions in BV-2 cells is strongly suggested.

## Conclusion

**Fig. 9:**
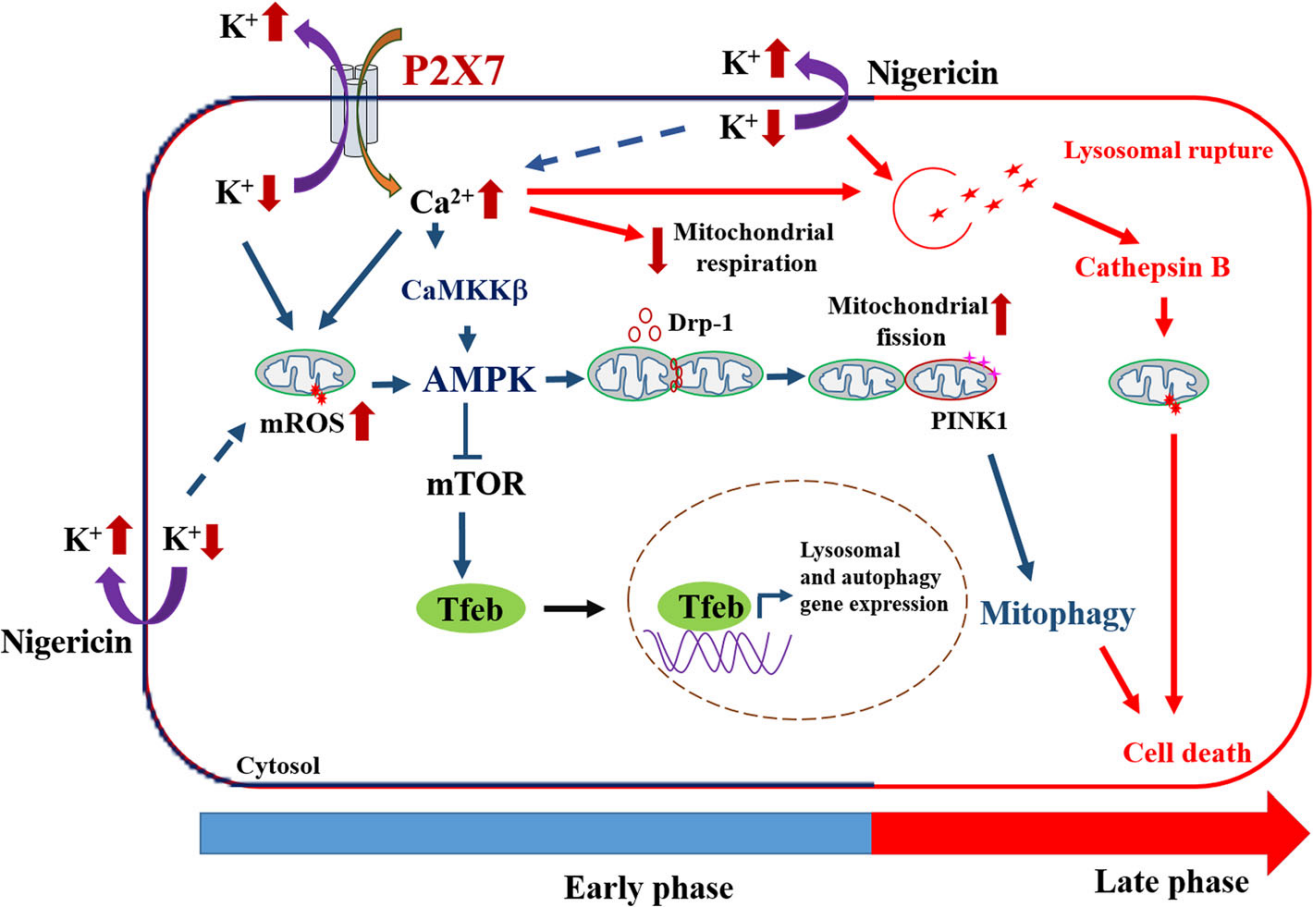
Schematic representation of P2X7 signaling pathways that lead to mitophagy, mitochondrial dysfunction, lysosomal biogenesis, and cell death in microglial cells

Taken together, our findings indicate the time-dependent actions of P2X7 in regulating the functions of mitochondria and lysosomes in microglial cells (Fig. 9). In early phase shortly after P2X7 activation, the Ca^2+^ influx and K^+^ efflux efficiently act as positive regulators for mitophagy and lysosomal biogenesis, but a negative modulator for mitochondrial respiration. A prolonged action of P2X7 signaling would lead to lysosomal destabilization and cell death. We demonstrate for the first time that P2X7 activation can rapidly trigger AMPK activation via ROS and CaMKKII pathways, which lead to mitochondrial fission, mitophagy induction and TFEB activation. However, inhibition of mitochondrial respiration and induction of lysosomal destabilization caused by P2X7 activation is independent of AMPK.

## Additional files

Additional file 1: **Figure S1.** Immunoblotting data of main Fig. 1a-c were quantified using Image J software. Data were the mean ± S.E.M. from 3 independent experiments. *p < 0.05, indicating the enhanced effects of ATP, BzATP and nigericin; #*p* < 0.05, indicating the antagonist effects of A438079 and/or P2X7^−/−^ on the individual action of ATP and BzATP. (TIF 3316 kb)

Additional file 2: **Figure S2.** Immunoblotting data of main Fig. 2c, d and f were quantified using Image J software and shown in Additional file 2: Figure S2A, 2B and 2C, respectively. Data were the mean ± S.E.M. from 3 independent experiments. *p < 0.05, indicating the significant effects of BzATP and nigericin; #*p* < 0.05, indicating the antagonist effects of mitoTEMPO and STO-609 on the action of BzATP and nigericin. (TIF 2618 kb)

Additional file 3: **Figure S3.** Immunoblotting analysis of main Fig. 3 were quantified using Image J software. Data were the mean ± S.E.M. from 3 independent experiments. *p < 0.05, indicating the significant effects of ATP, BzATP and nigericin; #*p* < 0.05, indicating the antagonist effects of A438079, mitoTEMPO, Compound C, siAMPK and P2X7^−/−^ on the individual action of ATP, BzATP and/or nigericin. (TIF 5179 kb)

Additional file 4: **Figure S4.** Immunoblotting analysis of main Fig. 5 were quantified using Image J software. Data were the mean ± S.E.M. from 3 independent experiments. *p < 0.05, indicating the significant effects of BzATP, nigericin and A769662; #*p* < 0.05, indicating the antagonist effect of siAMPK on the individual action of BzATP and nigericin. (TIF 4890 kb)

## Abbreviations

[Ca^2+^]i: Intracellular calcium
AMPK: 5’ AMP-activated protein kinase
ATP: Adenosine 5′-triphosphate
BMDM: Bone marrow-derived macrophages
BzATP: 2′(3′)-O-(4-Benzoylbenzoyl)adenosine 5′-triphosphate triethylammonium salt
CC: Compound C
DAMP: Damage associated molecular pattern
DMEM: Dulbecco Modified Eagle Medium
Drp-1: Dynamin-related protein-1
FACS: Fluorescence-activated cell sorting
FBS: Fetal bovine serum
FCCP: Carbonyl cyanide-p-trifluoromethoxyphenylhydrazone
OCR: Oxygen consumption rate
PBS: Phosphate-buffered saline
ROS: Reactive oxygen species
