# Supplementary figures and images for: AMPK-dependent and independent actions of P2X7 in regulation of mitochondrial and lysosomal functions in microglia
Source: Cell Commun Signal. 2018 Nov 20;16:83. doi: 10.1186/s12964-018-0293-3 (PMC6245559; doi:10.1186/s12964-018-0293-3)

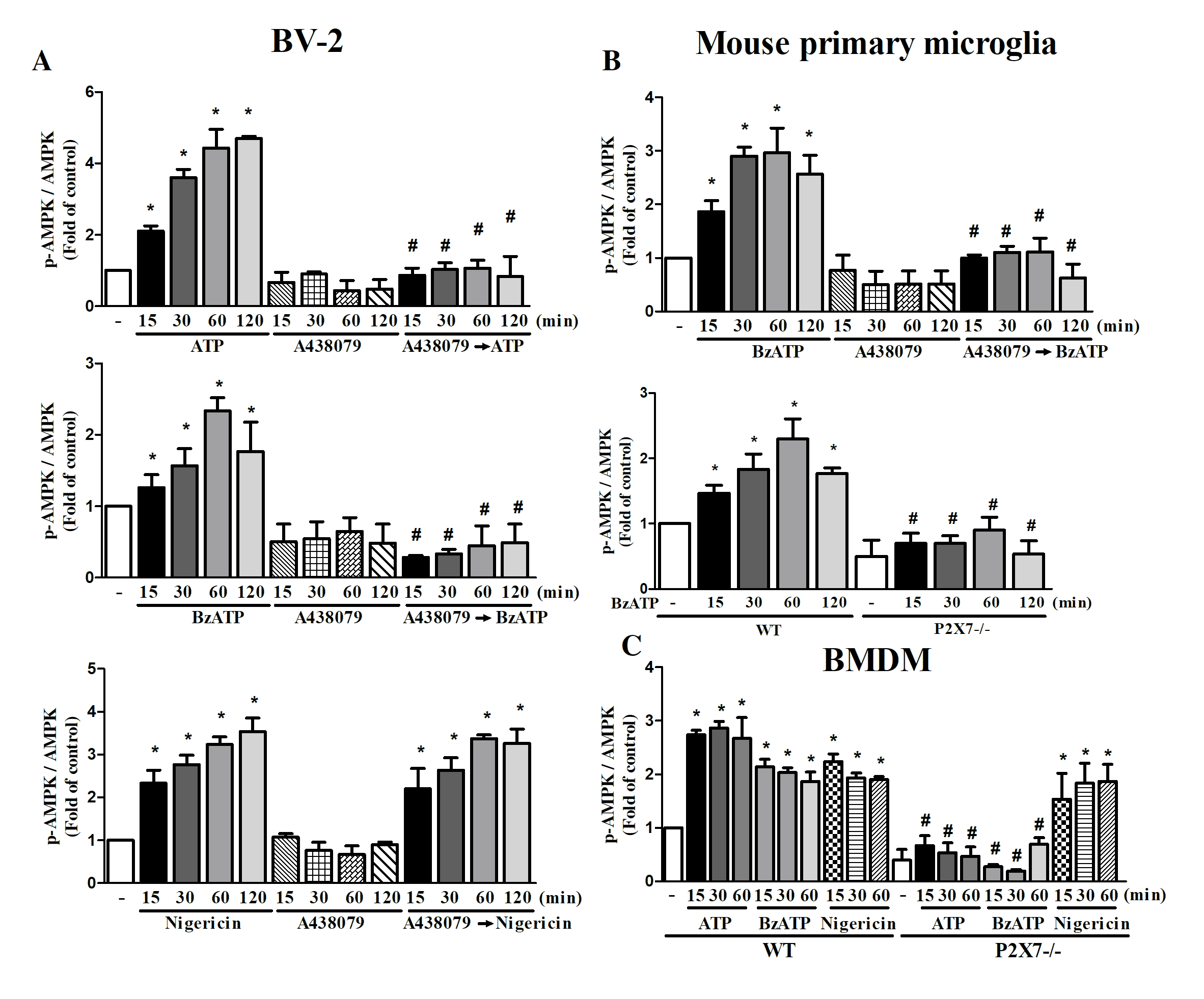

Supplement: Supplementary file 1 — Figure S1. Immunoblotting data of main Fig. 1a-c were quantified using Image J software. Data were the mean ± S.E.M. from 3 independent experiments. *p < 0.05, indicating the enhanced effects of ATP, BzATP and nigericin; #p < 0.05, indicating the antagonist effects of A438079 and/or P2X7−/− on the individual action of ATP and BzATP. (TIF 3316 kb) [file 12964_2018_293_MOESM1_ESM.tif]

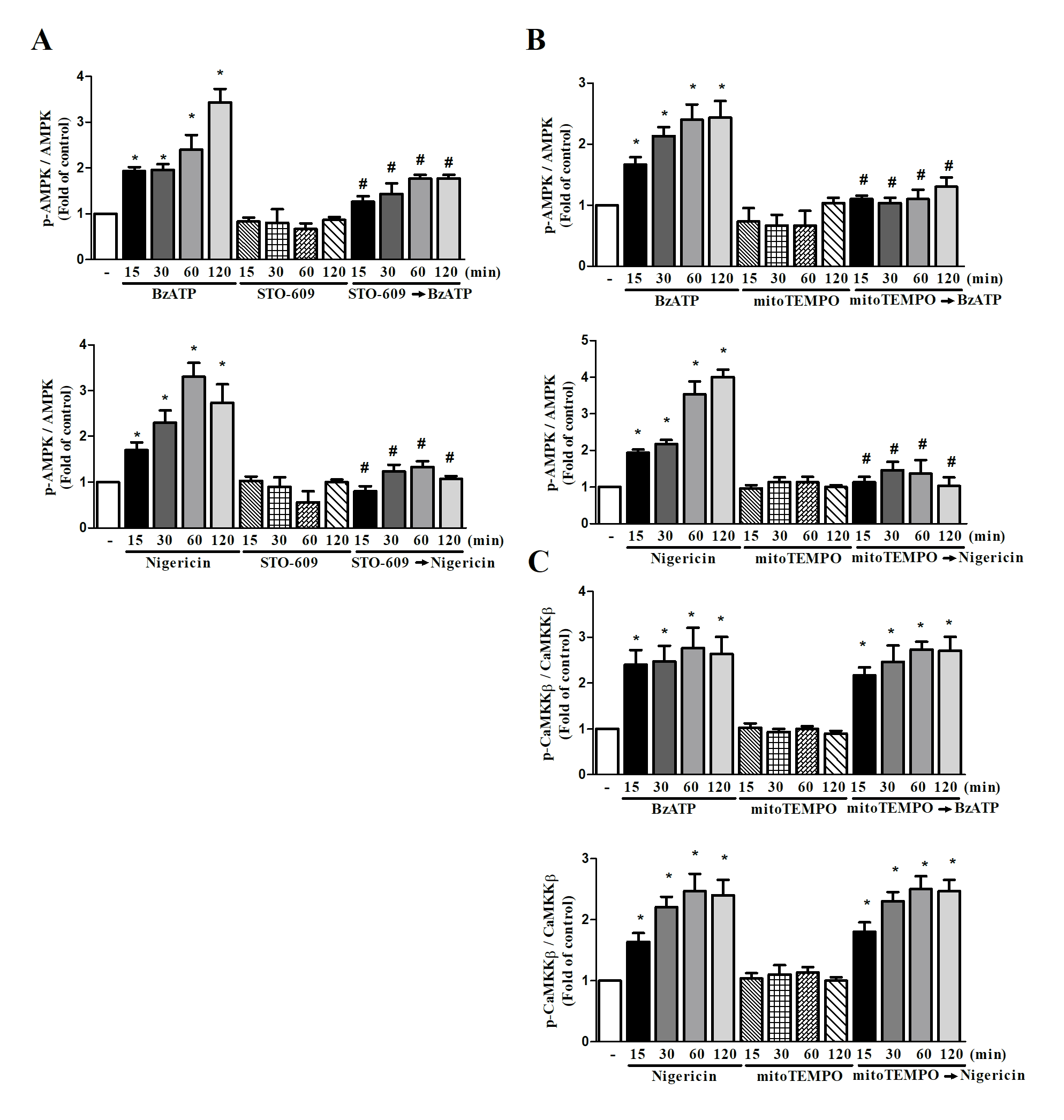

Supplement: Supplementary file 2 — Figure S2. Immunoblotting data of main Fig. 2c, d and f were quantified using Image J software and shown in Additional file 2: Figure S2A, 2B and 2C, respectively. Data were the mean ± S.E.M. from 3 independent experiments. *p < 0.05, indicating the significant effects of BzATP and nigericin; #p < 0.05, indicating the antagonist effects of mitoTEMPO and STO-609 on the action of BzATP and nigericin. (TIF 2618 kb) [file 12964_2018_293_MOESM2_ESM.tif]

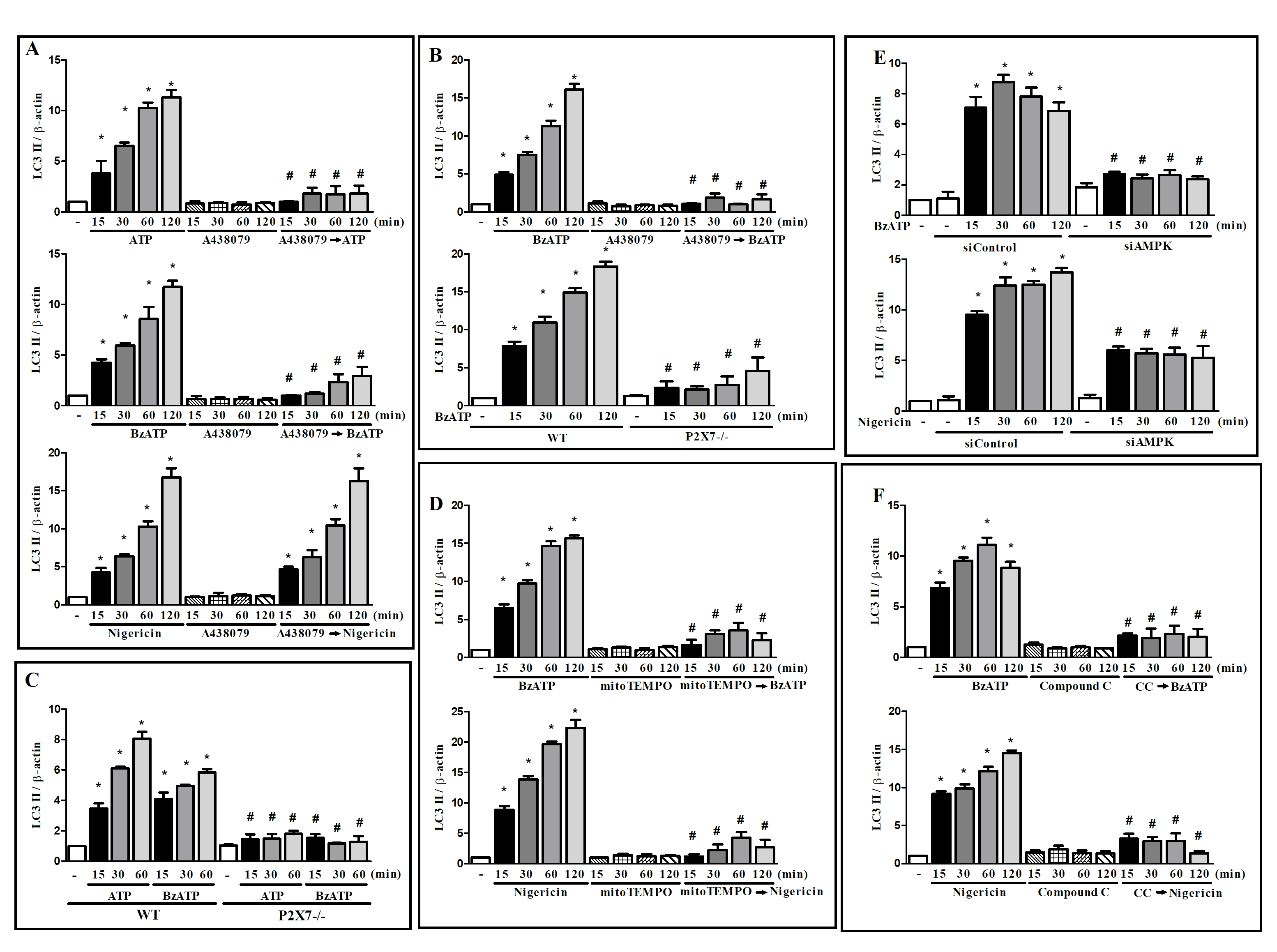

Supplement: Supplementary file 3 — Figure S3. Immunoblotting analysis of main Fig. 3 were quantified using Image J software. Data were the mean ± S.E.M. from 3 independent experiments. *p < 0.05, indicating the significant effects of ATP, BzATP and nigericin; #p < 0.05, indicating the antagonist effects of A438079, mitoTEMPO, Compound C, siAMPK and P2X7−/− on the individual action of ATP, BzATP and/or nigericin. (TIF 5179 kb) [file 12964_2018_293_MOESM3_ESM.tif]

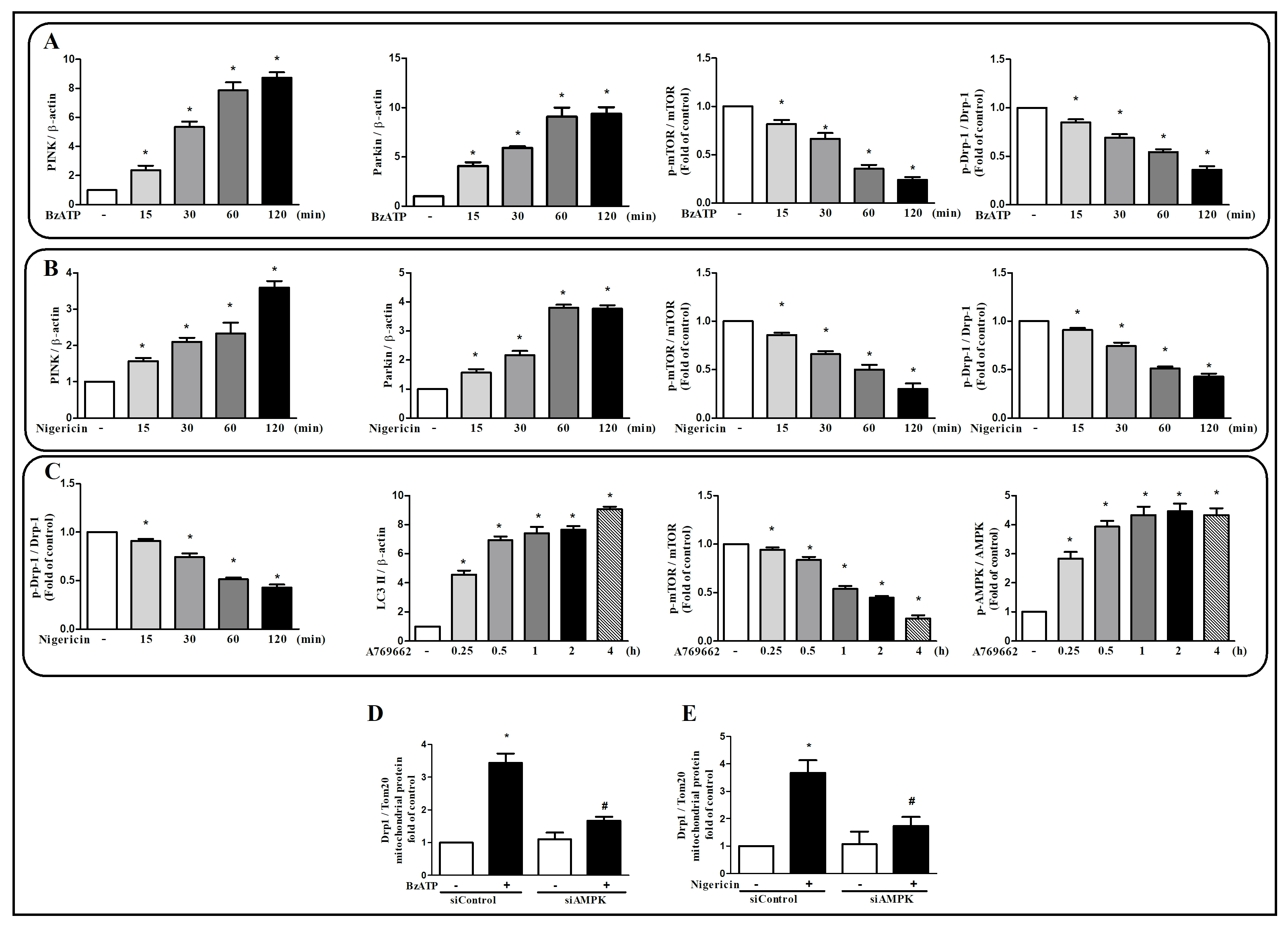

Supplement: Supplementary file 4 — Figure S4. Immunoblotting analysis of main Fig. 5 were quantified using Image J software. Data were the mean ± S.E.M. from 3 independent experiments. *p < 0.05, indicating the significant effects of BzATP, nigericin and A769662; #p < 0.05, indicating the antagonist effect of siAMPK on the individual action of BzATP and nigericin. (TIF 4890 kb) [file 12964_2018_293_MOESM4_ESM.tif]
